# Correlation of Serum Androgens and Pituitary Hormone Levels with Serum PSA Less Than 2.5 NG/ML

**DOI:** 10.1100/tsw.2007.177

**Published:** 2007-07-27

**Authors:** Mustafa Sofikerim, Özgür Oruç, Sadettin Eskicorapcı, Fuad Guliyev, Haluk Özen

**Affiliations:** Hacettepe University, School of Medicine, Department of Urology, Ankara, Turkey

**Keywords:** prostate specific antigen, testosterone, follicle-stimulating hormone, luteinizing hormone

## Abstract

The aim of this clinical study was to determine whether there is a relationship between total serum testosterone, free testosterone, FSH (Follicle-Stimulating Hormone), LH (Luteinizing Hormone) and serum prostate specific antigen (PSA) levels. We postulated that such a correlation existed then the use of hormone specific reference ranges might enhance the usefullness of PSA concentrations <2.5 ng/mL as a marker for prostate cancer.

Prior to digital rectal examination, serum was obtained from all patients between 8.30-10:00 AM for hormone and PSA concentrations. The study was performed on 210 male patients >40 years of age visiting our urology outpatient clinics. PSA was correlated to age (r = 0.23, p = 0.019), but there none between serum testosterone and age. No significant correlation was noted between testosterone or free testosterone and serum PSA levels, and none between serum FSH or LH and PSA. In age specific reference groups (41-49; 50-59; 60-69 years), we found no significant correlation between PSA and hormone concentrations.

In this population of eugonadal men with serum PSA values less than 2.5 ng/ml, serum androgens and pituitary hormones do not appear to correlate with serum PSA.

## INTRODUCTION

Serum prostate specific antigen (PSA) is widely used in screening, staging and/or monitoring prostate cancer. However, because other conditions can also affect serum PSA, significant false positive and false negative rates are associated with its use as a marker for cancer. The lower limit at which serum PSA can detect prostate cancer is not exactly known. A total PSA level of 2.6-4 ng/mL was associated with a 20.2% risk of prostate[1]. Biopsy-detected prostate cancer, including high-grade cancer, is not unusual among men with a total PSA of ≤4 ng/mL[2]. Many recent studies have suggested that a PSA cutoff point of 2.5 ng/mL may be more appropriate for screening; however, there is an 18.7% incidence of prostate cancer with PSA <2.0 ng/mL[3]. For screening prostate cancer, the PSA cutoff value is taken as 2.5

ng/mL in our clinic and others[2,3]. As a result, many men now present with benign digital rectal examination and serum total PSA levels <2.5 ng/mL. Despite strong circumferential evidence indicating that testosterone and pituitary hormones play an important role in the etiology of prostate cancer, most of prior prospective studies of hormones and prostate cancer shown no significant associations between hormones and prostate cancer risk[4,5,6,7,8,9]. On the other hand two studies have found a decreased risk of having prostate cancer with high levels of circulating total testosterone[10,11]. Hilz et al have found a slight reduction of average serum LH in prostatic cancer compared to benign prostatic hyperplasia (BPH)[12]. Serum FSH level was found to be higher for the patients with prostate cancer than the patients with BPH[11]. We designed this study to cast light on the possibility that hormone specific PSA reference ranges might enhance the usefulness of PSA below 2.5 ng/mL as a marker for prostate cancer, given that androgens and pituitary hormones affect PSA.

## PATIENTS AND METHODS

In this clinical study we evaluated, between February and December 2005, 210 male patients >40 years of age who visited our urology outpatient clinics. We excluded patients diagnosed as prostate cancer previously and who were receiving 5α reductase inhibitors, LHRH (Leutinizing Hormone Releasing Hormone) analogues, androgen receptor blockers and/or testosterone replacement therapy. Patients with serum PSA levels >2.5 ng/ml and/or abnormal digital rectal examination, who were selected as candidates for transrectal ultrasonography (TRUS) guided prostate biopsy, were also excluded. Before digital rectal examination, serum was obtained from an ante-cubital vein between 8.30-10:00 AM for measuring serum LH, FSH, testosterone and free testosterone levels. Serum total and free testosterone, FSH, and LH levels were measured by radioimmunoassay methods from fresh serum samples (Immulite 2000 Total Testosterone. solid phase. Chemiluminescent Enzyme Immunoassay. Diagnostic Products Corp, USA. Active Free Testosterone. RIA DSL-4900. Diagnostic System Laboratories, USA. Architest FSH, Architest LH, Chemiluminescent Microparticle Immunoassay, Abbott Laboratories, USA). Serum PSA levels were measured by The Elecsys total-free PSA Immunoassay (Roche Diagnostics GmbH. USA).

In parallel to Morgenthaler et al and Hoffman et al, patients with a serum total testosterone value of ≤ 300 ng/dl and free testosterone level of ≤ 9 pg/ml were classified as having low serum total testosterone and free testosterone[13,14].

Personal computer based Statistical Package for Social Sciences 13.0 (SPSS) for Windows was used for all statistical analysis to calculate the median and range. Percent free PSA was calculated by dividing free PSA concentration by total PSA. Serum hormone levels and corresponding PSA levels were then analysed using Pearson’s correlation coefficients. Correlations between serum hormone levels and corresponding PSA levels per age decade (age groups; 41-49 years. 50-59 years. 60-69 years) were done using Kruskal-Wallis One Way Analysis of Variance on Ranks (Multiple Comparison Procedures - Dunn's Method) test.

All protocols, including the informed consent procedures, were approved by our Institutional Review Board.

## RESULTS

The mean age was 54.2 years (range 41-69 years; n=210). The PSA range was 0.22 - 2.5 ng/mL with a mean of 1.2±0.7 ng/mL (Table I). Free PSA range was 0.05 -1.38 ng/mL, with a mean of 0.37±0.25 ng/mL. Free PSA% varied from 0.06 to 0.73 with a mean of 0.34±0.14. Serum testosterone varied from 182 to 1103 ng/dL (mean 534±169) with 92% located within the normal range. The mean serum free testosterone level was 10.17±3.7 pg/mL (range 1.9-21.6) with located 64% within normal range. The mean serum FSH and LH levels were 6.24±4.88 mIU/mL (range 1.37-29.9) and 5.29 ± 2.96 mIU/mL (range 1.39-18.36) respectively.

**TABLE I**
**Principal Clinical and Endocrinological Data**

| Parameter | Mean±SD | Range |
|---|---|---|
| Age (year) | 54.2 | 41-69 |
| PSA (ng/mL) | 1.2 ± 0.7 | 0.22-2.5 |
| Free PSA (ng/mL) | 0.37±0.25 | 0.05-1.38 |
| Percent free PSA | 0.34±0.14 | 0.06-0.73 |
| Testosterone (ng/dL) | 534 ± 169 | 182-1103 |
| Free Testosterone (pg/mL) | 10.17 ± 3.7 | 1.9-21.6 |
| FSH (mIU/mL) | 6.24 ± 4.88 | 1.37-29.9 |
| LH (mIU/mL) | 5.29 ± 2.96 | 1.39-18.36 |

FSH= Follicle stimulating hormone; LH= Luteinizing Hormone; PSA= Prostate specific antigen; SD= Standard deviation

Age and PSA concentrations were correlated significantly (r= 0.23. p= 0.019), but there was no significant correlation between free PSA, % free PSA and age. No significant correlation was found between testosterone, free testosterone and age or between age and LH concentration. A significant correlation was found between increasing age and FSH (r= 0.207. p= 0.034). There was an increase in the gonadotropins LH and FSH per age decade, with the most significant changes seen in individuals 60 years or older. Age related endocrinological data is shown in Table II.

No significant correlation was noted between testosterone or free testosterone and serum PSA levels, nor between FSH, LH and serum PSA levels. There was no significant correlation between free PSA or % free PSA and serum testosterone, free testosterone, LH and FSH levels. Table III shows correlation between clinical and endocrinologic parameters.

Within specific age (41-49, 50-59, 60-69 years), there was no significant correlation between PSA, free PSA, % free PSA and hormone concentrations.

**TABLE II**
**Age related changes of testosterone, free testosterone, FSH and LH**

| Age (Year) (n) | FSH (mIU/mL) | LH (mIU/ML) | Testosterone (ng/dL0 | Free Tesosterone (pg/mL) |
|---|---|---|---|---|
| **41-49 (66)** | **3.69** [a] (2.69-5.97) | **4.01** [a] (2.72-5.81) | 561[c] (407.5-671.5)[d] | 10.36 (7.7-11.83) |
| **50-59 (94)** | 5.03[ab] (3.88-7.17) | 4.81 [ab] (3.42-7.17) | 522 (451-616) | 10.38 (8.1-12.13) |
| **60-69 (50)** | **7.81** [b] (4.59-5.73) | **5.21** [b] (4.01-7.59) | 516 (420-580) | 9.45 (6.99-11.49) |
| **p-value** | **p<0.05** | **p<0.05** | **p>0.05** | **p>0.05** |

FSH= Follicle stimulating hormone; LH= Luteinizing Hormone

[a,b] Multiple Comparison Procedures (Dunn's Method): same letters on values show statistical non-significance
[c]Median
[d] 25-75th percentile

**TABLE 3**
**Correlations Between Clinical and Endocrinological Parameters**

| Parameter | Testosterone | freeTestosterone | FSH | LH |
|---|---|---|---|---|
| **Age** | -0.11 | -0.112 | **0.207**[a] | 0.163 |
| **PSA** | 0.048 | 0.043 | 0.052 | 0.046 |
| **Free PSA** | 0.035 | 0.038 | -0.072 | -0.073 |
| **Percent Free PSA** | -0.001 | 0.027 | -0.118 | -0.123 |

FSH= Follicle stimulating hormone; LH= Luteinizing Hormone; PSA= Prostate specific antigen
Correlation coefficients are given; [a]Statistical significance ($p<0.05$)

## DISCUSSION

Many men now present with benign digital rectal examination and serum total PSA levels less than 2.5 ng/mL. Recent data from the Prostate Cancer Prevention Trial show a prostate cancer detection rate of 16.3% in men with a PSA level < 1.0 ng/mL, compared with 27.7% for men with an initial PSA level of 1.1 to 2.0 ng/mL and 39.2% for those with 2.1 to 3.0 ng/mL[15]. The most important limiting factor in the use of PSA screening is its low specificity, which leads to a substantial number of unnecessary prostate biopsies. In order to increase the specificity of PSA at lower concentrations, simultaneous measurement of serum androgens and pituitary hormone concentrations could theoretically be useful.

Androgens are necessary for prostatic development, maintenance and activity. Testosterone and dihydrotestosterone are the major androgens in serum and tissue respectively. Dihydrotestosterone, which is derived from testosterone, acts on the prostate via androgen receptors (AR). The transactivation of AR by dihydrotestesterone has been previously shown[16]. Testosterone may have an effect on PSA secretion, as suppression of prostate cancer growth by endocrine manipulation has previously been shown[17]. Serum PSA is also reduced by surgical or medical castration in metastatic prostate cancer. Various methods of androgen suppression such as androgen receptor blockade, 5α reductase inhibition and LHRH analogues reduce serum PSA concentrations[18,19,20]. These studies show a direct relationship between serum testosterone and PSA levels when testosterone is reduced to castration levels. There is a significant rise in mean serum PSA levels in hypogonadal men treated with testosterone replacement therapy[21]. Further, there was a positive correlation between serum testosterone, LH and PSA concentrations in a group of 132 normal boys throughout puberty [22]. These studies all discussed a possible relationship between serum androgens and pituitary hormones with prostate specific antigen.

We therefore decided to look for a possible correlation between serum PSA (and % free PSA) and serum androgen and pituitary hormones. Percent free PSA levels are of interest because cancer detection rates increased significantly when the free/total PSA was <0.15 and PSA <4 ng/mL[1]. Were such a correlation to exist then the use of hormone specific reference ranges might enhance the ability of PSA below 2.5 ng/mL to act as a marker for prostate cancer.

Potential weaknesses of our study include patient selection bias; 1) Patients included in the study may have altered PSA levels that this study focuses on a group presenting for urologic care and are likely more at risk than the average population for conditions which may affect PSA. But our aim was to focus on the possibility that hormone specific PSA reference ranges might enhance the usefulness of PSA below 2.5 ng/mL as many men now present with the widespread use of PSA. So the study mainly focussed on these sort of patients. 2) Prostatic biopsy was not performed, thus the incidence of prostate cancer in the study population is not known. 3) Further, given the variability of PSA and hormone levels it would have been nice to have several values but only one serum sample from each patient was taken and also follow up would be necessary for prostate cancer detection.

Our data appear can be compared to the few other studies on this subject. A positive correlation with increasing age and PSA levels has been observed in several previous studies[23,24,25]. The increase in the gonodotropins LH and FSH with age is probably due to an intrinsic age-dependent phenomenon[26]. However, no significant change in serum testosterone with increasing age was found in a previous study of 312 men with symptoms of lower urinary tract obstructon, in whom serum hormones and serum PSA were measured without a cutoff level[24]. We found no correlation between total testosterone and age in our study, although others have demonstrated a significant inverse relationship[27,28]. We found no significant correlation between testosterone, free testosterone and pituitary hormones in eugonadal men with serum PSA levels similiar to data of the Prostate Study Group of the Austrian Society of Urology[24]. Others have found no relation between serum total testosterone concentration and serum PSA[23]; no PSA cutoff level was taken, and patients with prostate cancer were included in the final analysis. Only serum testosterone levels were studied to define whether there is correlation with serum PSA levels, whereas we analyzed the relationship between free PSA and percent free PSA with serum hormone levels and found no significant correlations. Similarly, examining age specific reference groups did not yield any significant correlations.

We conclude that determination of serum testosterone; free testosterone and pituitary hormones will not significantly improve the use of PSA for prostate cancer detection in patients with low PSA levels. Further new studies are needed to evaluate their effect on prostate cancer detection.
